# Supplementary material for: Triacylglycerol stability limits futile cycles and inhibition of carbon capture in oil-accumulating leaves
Source: Plant Physiol. 2024 Mar 2;197(2):kiae121. doi: 10.1093/plphys/kiae121 (PMC11849776; doi:10.1093/plphys/kiae121)
Supplement: kiae121_Supplementary_Data [file kiae121_supplementary_data.pdf]

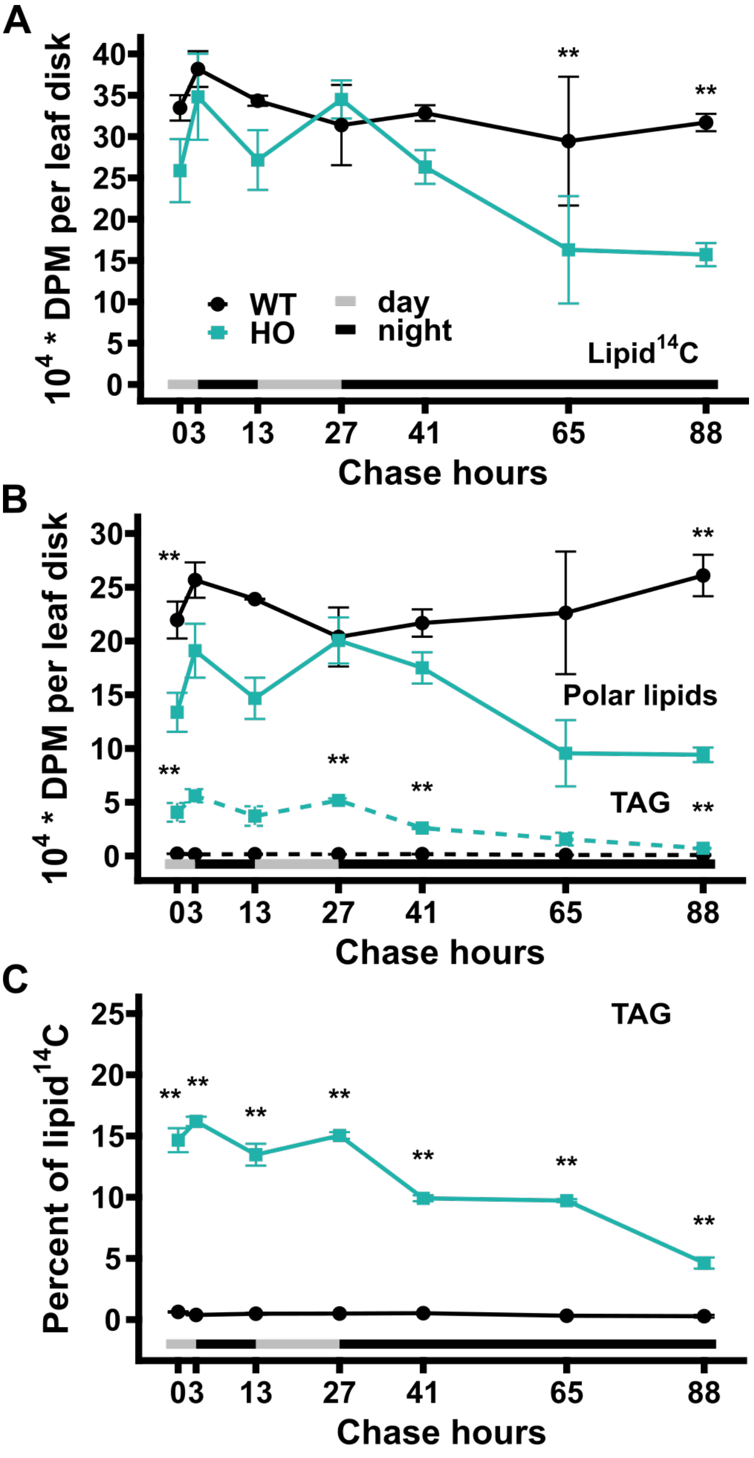

Supplemental Figure S1. $^{14}CO_2$ pulse-chase tracing of lipid metabolism in 62-day old WT and HO leaves under day-night cycles and constant darkness. Plants were pulsed with 1 mCi $^{14}CO_2$ for 1.5 hrs. At the pulse, the plants were grown for two day/night cycles, and at the end of the second night, they were kept in constant darkness. 20 mm diameter leaf disks were collected from each plant at each time point: 0 hrs, *n* = 6; 3-88 hrs, *n* = 2. (A) Quantitative analysis of total ^{14}C accumulation in leaf disks collected with 18 mm diameter cork bore. B) ^{14}C labeled Polar lipids. C) ^{14}C labeled TAG. For all panels, all data points are mean ± SE. Asterisks (*) indicating significant differences between lines (ANOVA and Tukey Honest Significant Differences test for multiple comparisons) p-value 0.05 – 0.01 = *, < 0.01 = **. WT: black lines and circles, HO: blue-green lines and squares. Abbreviations are disintegrations per minute (DPM) and triacylglycerol (TAG).

Supplemental methods:

WT and HO tobacco plants were grown in Percival E-41HO growth chambers set at 16 h light/8 h dark, 26°C/22°C, and fluorescent white light intensity at pot level across the chamber was 300–400 µmol photons m−2. s−1. Pots were watered three times a week, with one watering per week replaced by Peters 20/20/20 NPK fertilizer at 0.97 g/L. The pulse chase was as described in the main text with the following exceptions: 1) the pulse of $^{14}CO_2$ was 1.5 hrs in duration; 2) the chase was modified to include extended darkness from hrs 27-88; 3) no formic acid was added to extraction solvent for ^{14}C lipids; 4) 18 mm diameter leaf disks were collected.

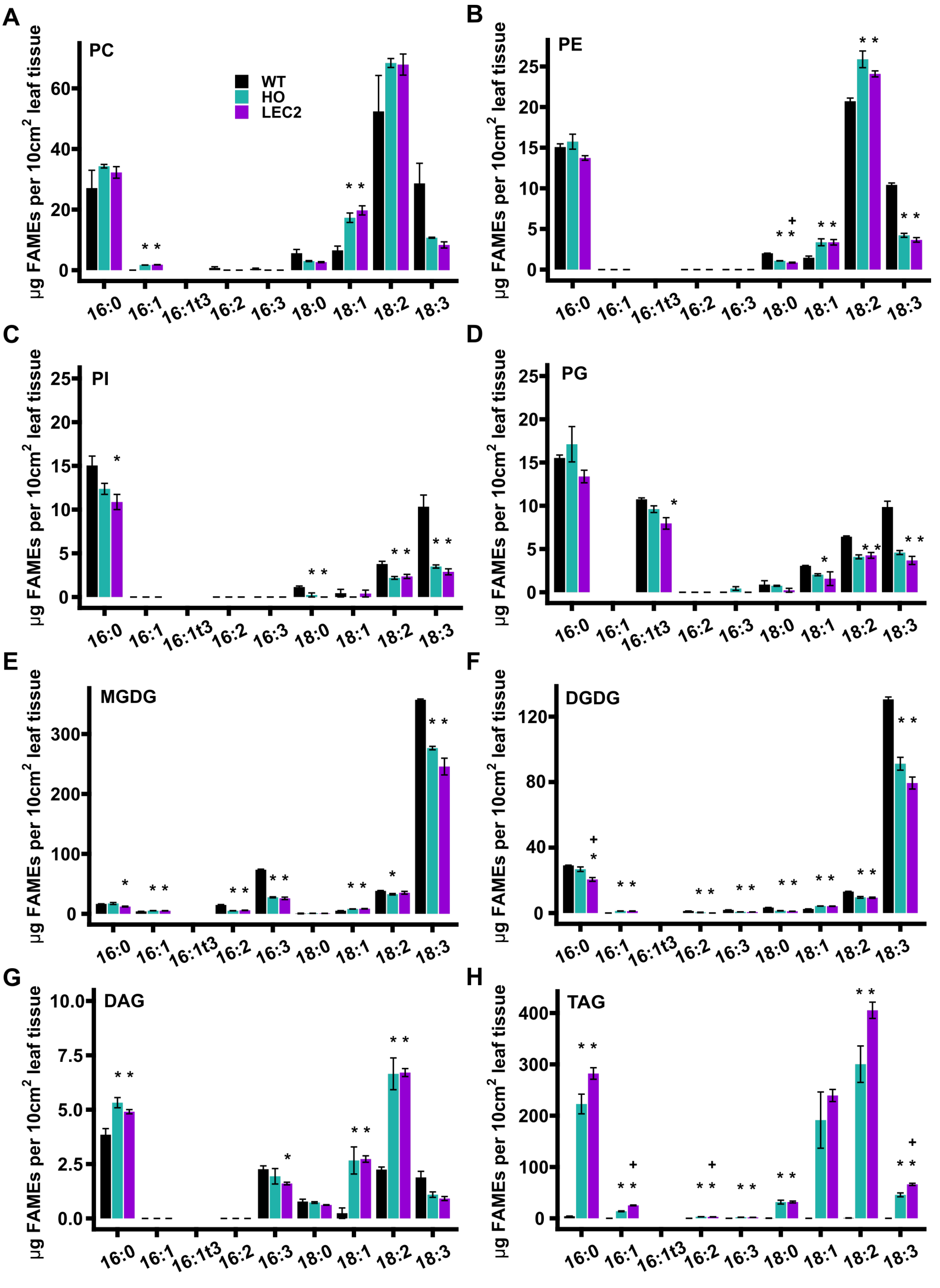

Supplemental Figure S2. **Fatty acid composition (micrograms per 10 cm^2 leaf area) of glycerolipids for wild-type (WT) and oil-accumulating (HO, LEC2) tobacco (Fig. 1B).** A cork bore (18 mm diameter) was used to collect leaf disks from 40-day old plants. Five leaf disks were collected from various leaves at random from a single plant and combined for a single sample. Abbreviations are PC: phosphatidylcholine, PE: phosphatidylethanolamine, PI: phosphatidylinositol, PG: phosphatidylglycerol, MGDG: monogalactosyldiacylglycerol, DGDG: digalactosyldiacylglycerol, DAG: diacylglycerol, TAG: triacylglycerol, and FAMEs: fatty acid methyl esters. The x-axis labels (X:Y) represent the number of carbons in the acyl chain (X) and the double bonds (Y). For all panels, bars represent the mean (n = 3, except LEC2 DAG which has n = 2) and error bars represent mean +/- one standard error. Asterisks (*) above HO and LEC2 bars indicate significant differences (p-value < 0.05) compared to WT (reference group). A plus sign (+) above LEC2 indicates significant differences between HO and LEC2.

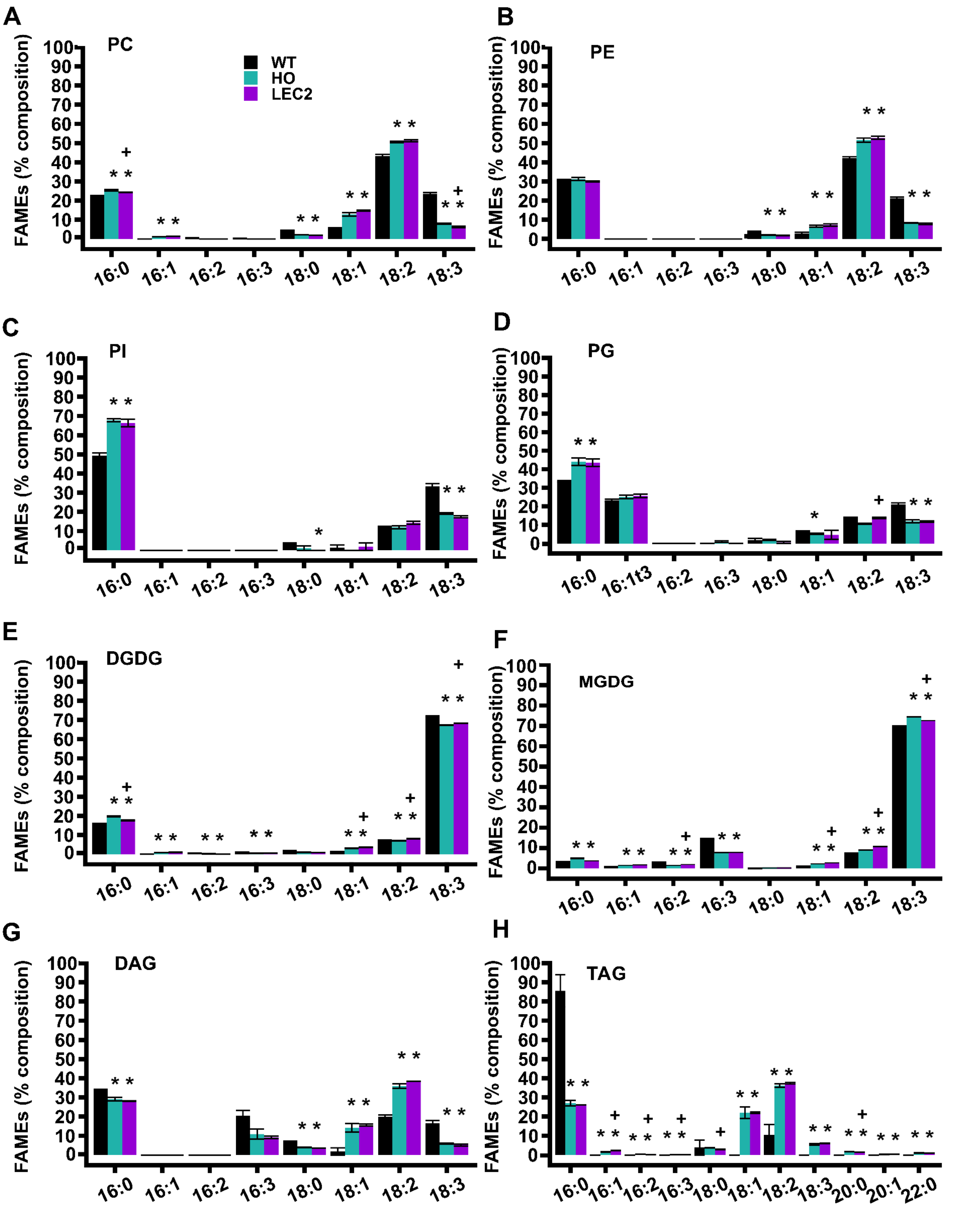

Supplemental Figure S3. **Fatty acid composition (percent of glycerolipid mass) of lipids for wild-type (WT) and oil-accumulating (HO, LEC2) tobacco (Fig. 1B).** A cork bore (18 mm diameter) was used to collect leaf disks from 40-day old plants. Five leaf disks were collected from various leaves at random from a single plant and combined for a single sample. Abbreviations are PC: phosphatidylcholine, PE: phosphatidylethanolamine, PI: phosphatidylinositol, PG: phosphatidylglycerol, MGDG: monogalactosyldiacylglycerol, DGDG: digalactosyldiacylglycerol, DAG: diacylglycerol, TAG: triacylglycerol , and FAMEs: fatty acid methyl esters.. The x-axis labels (X:Y) represent the number of carbons in the acyl chain (X) and the double bonds (Y). For all panels, bars represent the mean (n = 3, except LEC2 DAG which has n = 2) and error bars represent mean +/- one standard error. Asterisks (*) above HO and LEC2 bars indicate significant differences (p-value < 0.05) compared to WT (reference group). A plus sign (+) above LEC2 indicates significant differences between HO and LEC2.

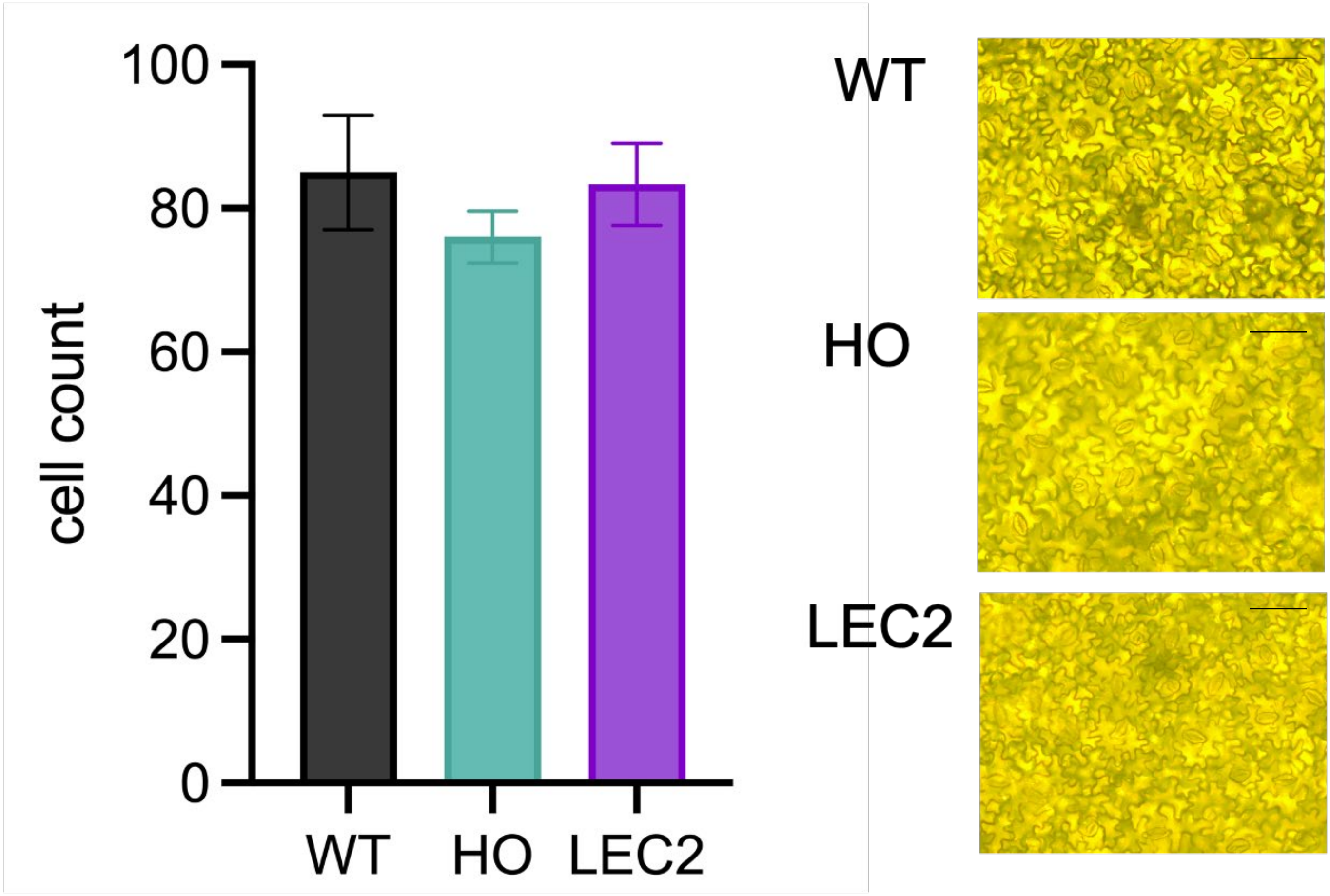

Supplemental Figure S4. Cell counts of leaf pavement cells from 45 day old WT, HO, and LEC2 tobacco at 20x magnification. Leaf disks were collected from the sixth and seventh leaf of each genotype (in the same manner as the labeling samples were collected) and imaged at 20x magnification to count the whole cells within the field of view. No significant differences were observed in the total of whole cells between the tobacco lines. Scale bar in the images represents 100 micrometers. All data points are mean ± SD of three biological replicates. Asterisks (*) indicating significant differences between lines, A: WT – HO; B: WT – LEC2; C: HO – LEC2 (ANOVA and Tukey Honest Significant Differences test for multiple comparisons) p-value 0.05 – 0.01 = *, < 0.01 = **.

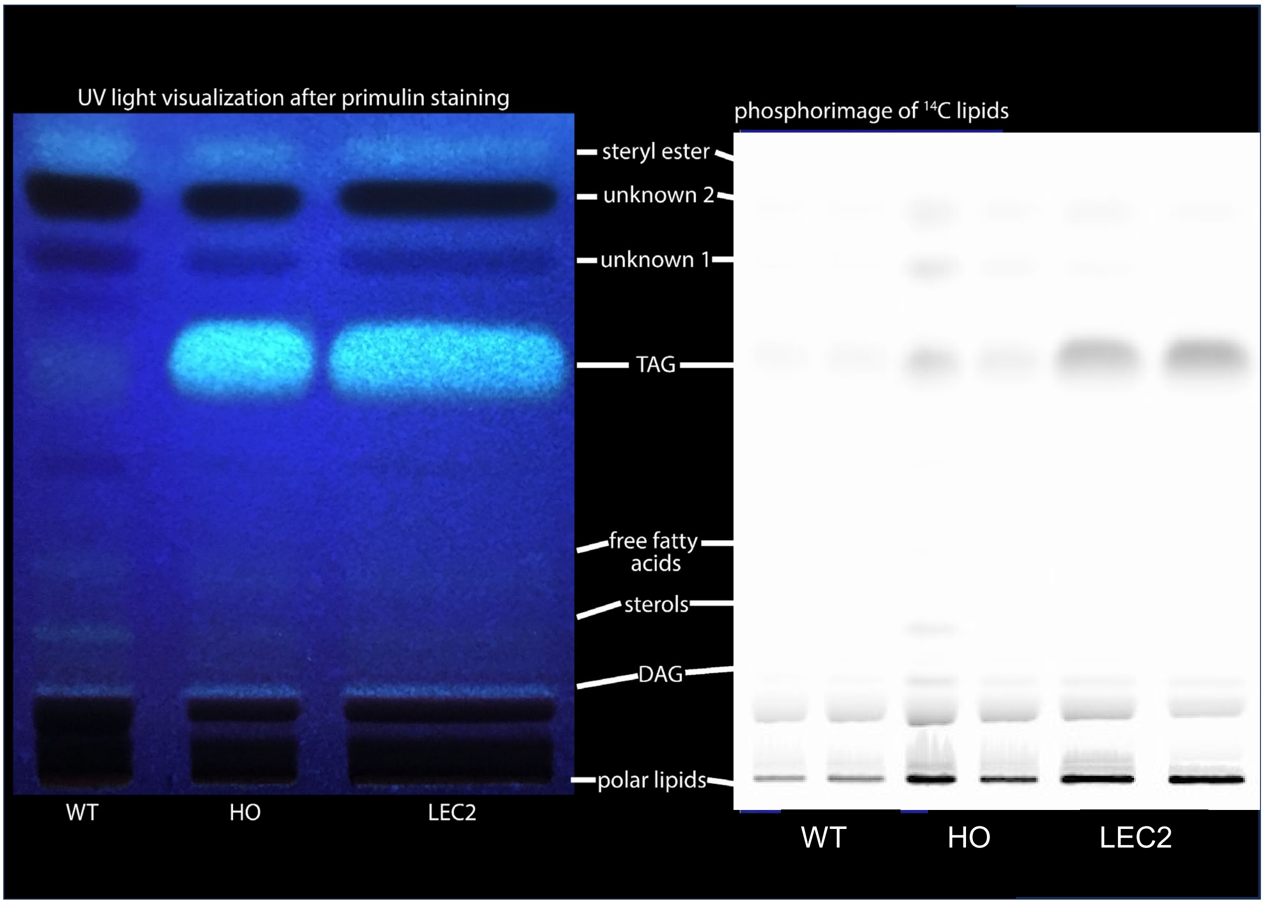

Supplemental Figure S5: Total lipid extract visualized after thin-layer chromatography (TLC) in hexane/diethyl ether/acetic acid (70/30/1, v/v/v). Representative images. The solvent system is optimized for neutral lipid separation. The amount of extract added to Analtech TLC plates was adjusted such that one centimeter contained approximately 400 micrograms of lipids. An aliquot of total lipid extract dissolved in chloroform was loaded to contain approximately 120,000 disintegrations per minute (DPM) of ^{14}C lipids for visualization of ^{14}C lipid bands on a phosphor reactive screen (24-48 hours of exposure) using a Typhoon 7000 phosphor imager (right image). Primulin staining (0.005 % primulin in acetone, w/v) verified adequate separation prior to exposing phosphor screens (left image). The primulin-stained TLC plate (left) was photographed during the separation of lipids in Fig. 1B, Supplemental Figures S2 and S3. The phosphorimage (right) was taken from the 30 hr time point of ^{14}C labeling of WT, HO, and LEC2 (Fig. 7, Supplemental Figure S5).

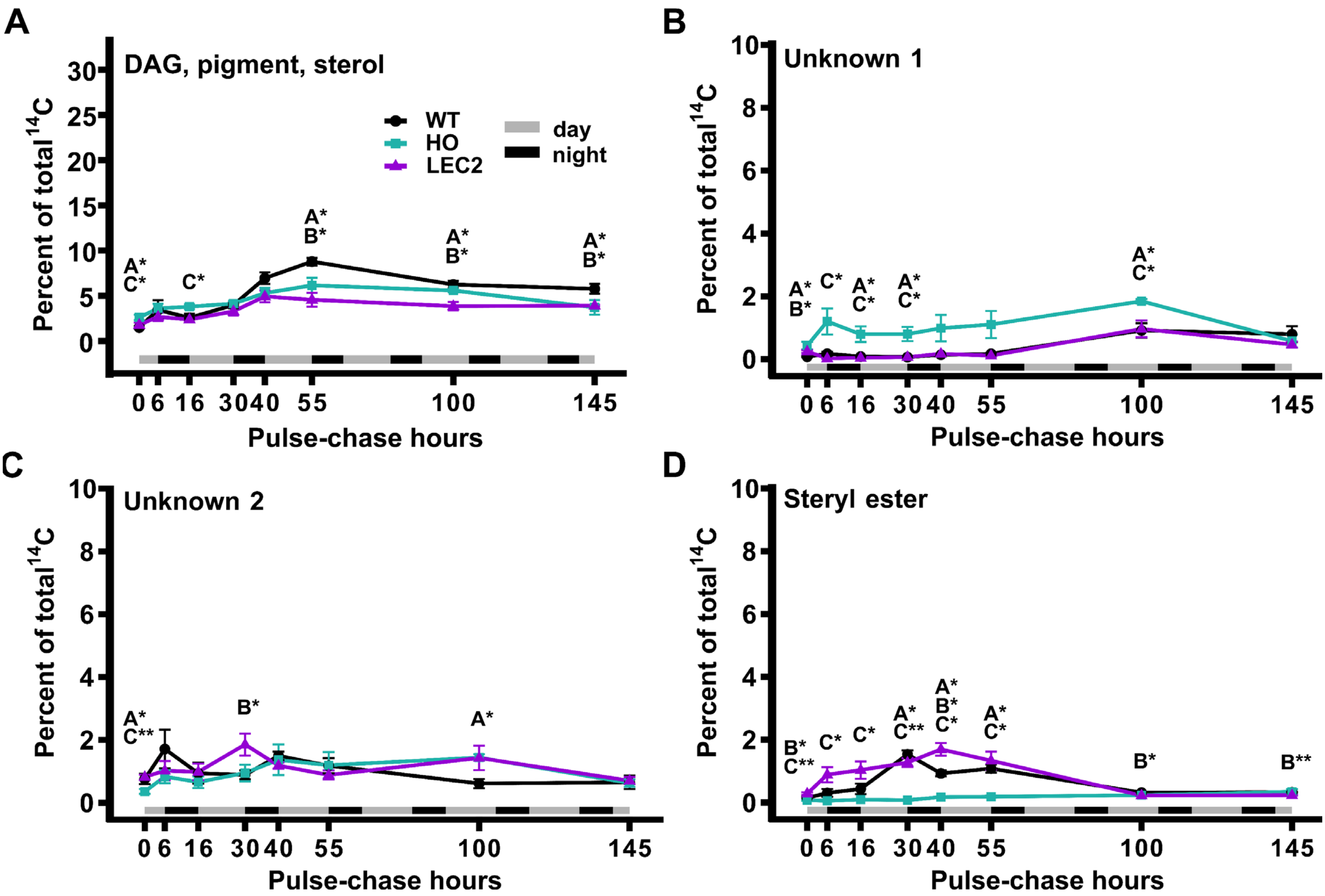

Supplemental Figure S6. Minor lipid fractions from the 145 hr $^{14}CO_2$ pulse-chase of WT, HO and LEC2 tobacco lines. The total lipid extract in Figure 5F is fractionated by TLC into: A) DAG (diacylglycerol), pigments, and sterols; B) Unknown 1; C) Unknown 2; and D) steryl ester. A total lipid extract (400 micrograms of total lipids) was separated via thin-layer chromatography in 70/30/1 hexanes/diethyl ether/acetic acid (v/v/v) and the amount of ^{14}C was quantified as a percentage of the total radioactivity. Standards of DAG, stigmasterol, and sitosterol verified the migration of lipid bands but bands could not be fully separated to resolve the amount of ^{14}C for individual bands. Unknowns 1 and 2 (B and C) migrated above TAG (triacylglycerol). For all panels, all data points are mean ± SE. Asterisks indicating significant differences between lines, A: WT – HO; B: WT – LEC2; C: HO – LEC2 (ANOVA and Tukey Honest Significant Differences test for multiple comparisons) p-value 0.05 – 0.01 = *, < 0.01 = **.
